# Response patterns in finger and central body skin temperatures under mild whole body cooling in an elderly and in a young male - a pre-study

**DOI:** 10.1186/2046-7648-4-S1-A32

**Published:** 2015-09-14

**Authors:** Kalev Kuklane, Leif Vanggaard, Juhani Smolander, Amitava Halder, Karin Lundgren, Chuansi Gao, Jari Viik, Jarmo Alametsä

**Affiliations:** 1 The Thermal Environment Laboratory, Division of Ergonomics and Aerosol Technology, Department of Design Sciences, Faculty of Engineering, Lund University, Lund, Sweden; 2 Danish Arctic Institute, Copenhagen, Denmark; 3 Tampere University of Technology, BioMediTech, Tampere, Finland

## Introduction

The arteriovenous anastomoses (AVAs) in distal body parts (especially hands and feet) and the peripheral veins draining them constitute the "AVA organ" that may provide around one third of the total skin surface area for heat exchange [1]. The temperatures over AVA-regulated skin sites are seldom taken into account and therefore actual heat loss may be underestimated, especially in studies employing mild whole-body cooling. The aim of this test was to study the response pattern of finger skin temperature (rich in AVAs) with non-AVA sites during transient whole-body cooling in an elderly and a young man.

## Methods

Two subjects, old (78 years, weight 73 kg, height 1.74 m) and young (31 years, weight 70 kg, height 171 cm) volunteered. Rectal temperature (every 10 s), skin temperature at 8 body sites and fingertips (every 10 s), pulse (every 15 s), cold discomfort/thermal sensation (10 min), IR-imaging (10 min), ballistocardiographic measurements with pressure-sensitive EMFi film sensor strips on the neck near carotid artery and on the right ankle, and a larger EMFi sensor on the seat beneath the measured person [2,3] were recorded. Instrumentation (20-25 minutes) was carried out in a chamber at about 29 °C. The subjects were dressed in shorts and seated on chair with arms supported at the heart level, and right upper arm wearing blood pressure band. Ambient temperature was set to 29 °C for 25 minutes and then gradually lowered at a mean rate of 0.13 °C.min^-1 ^for the next 100 minutes stabilizing at 17 °C. Mean (SD) air flow of 0.45 (0.14) m.s^-1 ^was directed into the back. After the cooling stabilized, bicycle ergometer exercise sessions (50 W) were added to observe the effect of exercise.

## Results and discussion

Classical area-weighted mean skin temperature in the old and in the young person did not show differences. Rectal temperature and body heat content in the old person reduced at a quicker rate than in the young one. The old subject reported less discomfort and cold sensation as compared to the young. The temperature in extremities (hands, feet = AVAs areas) dropped quicker and lower in the young subject than in the old. One major ballistocardiographic finding was that the systolic and diastolic amplitudes increased strongly, especially, with the older person reflecting the increased workload of the heart due to coldness. This was seen also in dramatically increased blood pressure values (diastolic 68-->101 and systolic 125-->176 mmHg). Skin rewarming with the older person seemed to be more gradual as seen in cold limbs in thermal images.

## Conclusion

The observations of this limited study indicate clear age-related differences in the peripheral circulatory response to a mild cold challenge. The peripheral circulatory response may be associated with the normal aging process. If the present result holds true with a larger, and with a significant number of older people, then these findings might open up avenues to develop 'smart clothes' applications for the elderly population to compensate ambient temperature changes.

## References

[B1] Taylor NAS Machado-Moreira CA van den Heuvel AMJ Caldwell JN Hands and feet: physiological insulators, radiators and evaporators European Journal of Applied Physiology 2014 114 10 2037 2060 10.1007/s00421-014-2940-8 25011493

[B2] Alametsä J Smolander J Vanggaard L Halder A Lundgren K Gao C Viik J Kuklane K Varheenmaa M Age-related circulatory responses to whole body cooling: observations by ballistocardiographic EMFi sensors Proceedings of Ambience14&10i3m, Scientific Conference for Smart and Functional Textiles, Well-Being, Thermal Comfort in Clothing, Design, Thermal Manikins and Modelling, 7-9 September 2014 Tampere University of Technology, Tampere, Finland

[B3] Alametsä J Kuklane K Smolander J Vanggaard L Halder A Lundgren K Gao C Viik J Age-related circulatory responses to whole body cooling: observations by heart rate variability Finnish Journal of eHealth and eWelfare 2015 7 2-3 57 64

